# Different models of genetic variation and their effect on genomic evaluation

**DOI:** 10.1186/1297-9686-43-18

**Published:** 2011-05-17

**Authors:** Samuel A Clark, John M Hickey, Julius HJ van der Werf

**Affiliations:** 1 School of Environmental and Rural Science, University of New England, Armidale, NSW, 2351, Australia; 2 Cooperative Research Centre for Sheep Industry Innovation, Armidale, NSW, 2351, Australia

## Abstract

**Background:**

The theory of genomic selection is based on the prediction of the effects of quantitative trait loci (QTL) in linkage disequilibrium (LD) with markers. However, there is increasing evidence that genomic selection also relies on "relationships" between individuals to accurately predict genetic values. Therefore, a better understanding of what genomic selection actually predicts is relevant so that appropriate methods of analysis are used in genomic evaluations.

**Methods:**

Simulation was used to compare the performance of estimates of breeding values based on pedigree relationships (Best Linear Unbiased Prediction, BLUP), genomic relationships (gBLUP), and based on a Bayesian variable selection model (Bayes B) to estimate breeding values under a range of different underlying models of genetic variation. The effects of different marker densities and varying animal relationships were also examined.

**Results:**

This study shows that genomic selection methods can predict a proportion of the additive genetic value when genetic variation is controlled by common quantitative trait loci (QTL model), rare loci (rare variant model), all loci (infinitesimal model) and a random association (a polygenic model). The Bayes B method was able to estimate breeding values more accurately than gBLUP under the QTL and rare variant models, for the alternative marker densities and reference populations. The Bayes B and gBLUP methods had similar accuracies under the infinitesimal model.

**Conclusions:**

Our results suggest that Bayes B is superior to gBLUP to estimate breeding values from genomic data. The underlying model of genetic variation greatly affects the predictive ability of genomic selection methods, and the superiority of Bayes B over gBLUP is highly dependent on the presence of large QTL effects. The use of SNP sequence data will outperform the less dense marker panels. However, the size and distribution of QTL effects and the size of reference populations still greatly influence the effectiveness of using sequence data for genomic prediction.

## Background

Genomic selection (GS) is a method to predict breeding values in livestock; however the underlying mechanism by which it predicts is not fully clear. The initial premise of GS was that it was based on the predicted effects of quantitative trait loci (QTL) in linkage disequilibrium (LD) with markers [1]. However, there is increasing evidence that GS also relies on "relationships" between individuals to accurately predict genetic values [2], because genomic predictions are more accurate when predicted individuals are more closely related to a reference population.

Given this debate, a better understanding of what GS is actually predicting is relevant for several reasons. First, the LD/QTL paradigm suggests that accurate predictions of breeding values will persist for several generations into the future allowing for a reduced number of phenotypic measurements [3]. Furthermore, it assumes that higher marker densities may allow for the prediction of breeding values across breeds [4] In contrast, if the relationship paradigm is true, then the predictive ability based on genomic data would persist only for one or two generations ahead. Therefore, continuous measurements of phenotypes of individuals that are related to selection candidates would be needed.

The LD/QTL model has been further challenged by the observation that for many traits only a small part of the additive genetic variance is explained by variation at known QTL [5,6]. Consequently, Fearnhead et al. [7] noted that inconsistencies often exist between high estimates of heritability and the small proportion of total genetic variance explained by QTL and they proposed that a rare variant model might explain this "missing heritability". These results from whole-genome analysis studies have raised questions about the true model underlying (quantitative) genetic variation which is still largely unknown.

The potential models underlying additive genetic variation range from an infinitesimal model based on the action of very many genes, each with a very small effect [8] to a model based on a small number of genes having a large effect and many genes having a near zero effect (QTL model). Although experimental data is needed to provide more evidence about the true model underlying genetic variation, simulation can be used to explore the behaviour of various prediction methods used in genomic selection.

Prediction methods vary in how much they allow individual loci to contribute to variation. The gBLUP method assumes equal variance across all loci [9]. In contrast, the Bayes B approach allows the marker loci to explain different amounts of variation, with only a small number of loci having an effect and many loci having no effect [1]. Therefore, each of these methods is expected to be suited to different models of variation. For example, gBLUP is expected to be suited to infinitesimal model assumptions and the Bayes B model is expected to be best suited to assumptions made by the QTL model. The question is whether the performance of each prediction method is dependent upon the true underlying genetic model, and whether these methods are robust against changes to the model of variation. Previously, it has been shown that while assuming the infinitesimal model over the short term, the traditional BLUP method (covariance defined by pedigree relationships) is quite robust against drastic deviations from that model [10]. Conversely, it is unknown how well the Bayes B method will perform when the true model of variation is more "infinitesimal".

The objectives of this research were to evaluate the accuracy and robustness of genomic methods used for genomic selection under various underlying genetic models and marker densities and for these various models to compare the accuracy of genomic selection when the validation individuals were one generation, several generations, or one sub-population removed from the prediction animals.

## Methods

### Base genotype simulations

Genotype simulations were conducted using the Markovian Coalescence Simulator (MaCS) [11] to simulate 1,000 base haplotypes. Thirty chromosomes each with base haplotypes of 100 cM (1 · 10^8 ^base pairs) were simulated with a per site mutation rate of 2.5 · 10^-8^. The total number of SNP segregating on the genome was approximately 1,670,000 (SNP sequence). Sixty thousand SNP markers and 5,000 SNP markers were randomly selected from all SNP in the genome sequence and these markers were used in the 60K and 5K analyses respectively. To give the simulation a realistic population structure, we simulated a population with an effective size of 100 and with historical Ne 1,000 years, 10,000 years and 100,000 years ago equal to 1,256, 4,350 and 43,500, respectively, which were loosely based on estimates by Villa-Angulo et al. [12] for Holstein cattle.

The base population haplotypes were randomly allocated to 200 base male and 1,000 base female animals of a simulated population structure, with 10 subsequent generations receiving these haplotypes via mendelian inheritance, allowing recombination to occur according to the genetic distance, i.e. 1% recombination frequency per cM. The pedigree was split into two divergent lines each with 10 generations and each generation containing 1,000 individuals i.e. 500 males and 500 females. Ten percent of the males were randomly selected and randomly mated to all females. Each female had two offspring per generation.

The different models used to simulate the additive genetic variation were: 1) the QTL model (QM) with 100, 1,000 and 10,000 QTL, 2) a rare variant model (RM) with 100 and 1,000 QTL, the infinitesimal model (IM) and a traditional polygenic model. Heritability (*h*^2^) for all models was 0.3.

### The QTL and the rare variant models

The true breeding value (*a*) of each animal was determined using:

where *β_j _*is the additive effect of QTL genotype (*j*) and *g_ij _*is the QTL genotype at locus *j *which is coded as 0, 1, or 2 and is the number of copies of the QTL that an individual (*i*) carries. Each QTL was randomly chosen from all segregating SNPs in the base generation.

For both the QM and RM, all of the genetic variance was explained by QTL. The effect of each QTL was drawn from a gamma distribution with a shape and scale of 0.4 and 1.66 respectively [1] and had a 50% chance of being positive or negative. All simulation parameters were common to both the QTL and rare variant models, however, under the RM all QTL were assigned to SNP markers with an allele frequency <0.01. Each SNP had a 3% chance of being used as a marker and a 0.05% chance of being used as a QTL.

### Infinitesimal model

The true breeding value (*a*) of each animal was again determined using:

where *β_j _*is the additive effect of genotype (*j*) and *g_ij _*is the genotype at locus *j *which is coded as 0, 1, or 2 and is the number of copies of the QTL that an individual (*i*) carries. All of the SNP in this model were given an effect drawn from a normal distribution and had a 50% chance of being positive or negative.

To ensure that the heritability of the QTL, rare variant and infinitesimal scenarios remained constant, the residual variance was scaled relative to the variance of the breeding values of individuals in the base generation, which was given by:

where ***a ***is a vector of breeding values of individuals in generation 1 and *n *is the number of individuals in that generation.

### The traditional polygenic model

The genetic values for the base individuals were simulated using a traditional polygenic simulation model which uses the formula:

where z is a random variable drawn from a standard normal distribution z~ N(1,0) and *σ_a _*is the genetic standard deviation. The breeding values for the subsequent generations were obtained using the following equation:

where *a_sj _*and *a_dj _*are the parental breeding values and *MS_i _*is a term for Mendelian sampling given by  where  is the average inbreeding coefficient of the parents of individual *i *and *V_a _*is the genetic variance.

### Statistical analyses and breeding value estimation

Three methods were used to estimate breeding values:

1) Bayes B as described by Meuwissen et al. [1], which uses a model that assumes that only a proportion of the loci explain the total genetic variance and that many markers explain zero variance. The statistical model for the implementation of Bayes B can be written as

where *y *is the phenotype of animal *i*, μ is the overall mean, *k *is the number of marker loci, *X_ij _*is the marker genotype at locus *j *which is coded as 0, 1, or 2 and is the number of copies of the SNP allele that individual (*i*) carries, *β_j _*is the allele substitution effect at locus *j*, *δ_j _*is a 0/1 variable indicating the absence (with probability π) or presence (with probability 1 - π) of locus *j *in the model, and *e_i _*is the random residual effect. The value for parameter π was 0.95. The genetic variance was fixed to the value resulting from the data simulation and the value for the residual variance was estimated from the data.

Marker effects *β_j _*were estimated by computing means of the posterior distribution resulting from a Monte Carlo Markov Chain (MCMC) and was implemented using AlphaBayes [13]. For each replicate within each scenario, a burn-in period of 20,000 cycles was used before saving samples from each of an additional 40,000 MCMC cycles, therefore using a total of 60,000 MCMC cycles.

The genomic estimated breeding value (GEBV) for animal *i *in the test set was estimated as:

where  is the mean effect at locus *j *obtained from the post-burn in samples.

2) gBLUP, which assumes an equal variance for each marker and uses a genomic relationships matrix among all individuals in a reference set and a test set allowing it to compute variance components and best linear unbiased predictions (BLUP) from a mixed model. This was achieved by replacing the pedigree-based relationship matrix with the genomic relationship matrix (G) estimated from SNP marker genotypes to define the covariance among breeding values. As in Hayes et al. [14], we assumed a model

where *y *is a vector of phenotypes, μ is the mean, 1*_n _*is a vector of 1s, Z is a design matrix allocating records to breeding values, *g *is a vector of breeding values for animals in the reference set and the test set and *e *is a vector of random normal deviates ~. Furthermore  where G is the genomic relationship matrix, and  is the genetic variance for this model. The genomic relationship matrix was formed as defined in VanRaden [15]; where M is the incidence matrix that specifies which alleles each individual inherited; the frequency of the second allele at locus *i *is *p_i_*, and the matrix P contains the allele frequencies expressed as a difference from 0.5 and multiplied by 2, such that column *i *of P is 2(*p_i _*- 0.5). Subtraction of P from M gives Z, which sets the expected value of *u *to 0. Subtraction of P gives more credit to rare alleles than to common alleles when calculating genomic relationships. Therefore G = ZZ'/[2∑*pi*(1 - *pi*)]. The division by 2∑*pi*(1 - *pi*) makes G analogous to the numerator relationship matrix (A).

3) Traditional BLUP which ignores genomic data and relies on information from ancestors using a numerator relationship matrix (A). This method uses the same model as gBLUP (above) however with the vector of additive genetic values *g *replaced by *a*, with  where A is the numerator relationship matrix and  is the additive genetic variance.

Variance components for both BLUP methods were estimated with ASREML [16] and the model solutions yielded estimated breeding values. The accuracy of the estimated breeding values in the test set was calculated as the correlation between estimated and true breeding values.

Three reference populations (2,000 individuals) were assigned to test the effect of varying the relationships between animals in the reference population and test population, each time using generation 10 of line 1 (1,000 individuals) as the test set. Reference set: 1) Generations 8 and 9 of line 1, were used to observe the effect of using closely related animals in the test and reference populations; 2) Generations 1 and 2 of line 1, were used to test divergent relationships; and 3) Generations 8 and 9 of line 2, were used to represent a different strain or closely related breed. Each method used phenotypes from the reference populations to estimate the breeding value of individuals in the test set. Eight replicates were performed and the estimated genetic values for each method were compared to the simulated true genetic values. The traditional BLUP method acted as a control using the entire pedigree, however only individuals from each respective reference population had phenotypes.

Whole-genome SNP sequence data was used for both genomic methods; gBLUP and Bayes B. Genotype data on all ~1.67 million SNPs were used and the Bayes B method was implemented with π = 0.998 so that a similar number of SNP were included in the model as with 60,000 markers, i.e. ~ 3,000. Average SNP effects were estimated in reference populations 1 and 2 to predict the genetic value of individuals in the 10^th ^generation of line 1. The gBLUP method was also implemented using SNP sequence data. A genomic relationship matrix was formed (as above) using all SNP on each chromosome, each separate matrix was then weighted according to the proportion of the total SNP to give an averaged whole-genome relationship matrix. Phenotypic data from animals in reference populations 1 and 2 were used to predict the genetic value of individuals in the 10^th ^generation of line 1.

## Results

The Bayes B method gave a more accurate prediction of breeding value than gBLUP and was robust against the changes to the underlying model of genetic variation. It had the highest accuracy of the estimated breeding value in both the QM and RM (Table 1). The highest accuracy was achieved by the Bayes B method when genetic variation was controlled by a few QTL with relatively large effects (100 QTL). Also under the RM, the Bayes B method gave a more accurate prediction of breeding value than gBLUP and BLUP especially when only a few QTL controlled variation. Although Bayes B was not significantly better than gBLUP under the 1,000 RM there was a distinct trend that Bayes B predicted breeding value more accurately than gBLUP. As the model of variation became more polygenic, the superiority of Bayes B decreased, however its predictive accuracy was not significantly different to that of gBLUP, even under the infinitesimal and polygenic models.

**Table 1:** The average accuracy of breeding value estimates (±SE) in the test set obtained from three methods of analysis of reference population 1 with 60,000 SNPs and different genetic models

Model	No. QTL	Bayes B	gBLUP	BLUP	Est. h^2 ^(Range) ^1^
**QM**	**100**	0.82 (0.007)	0.56 (0.017)	0.46 (0.023)	0.32 (0.29-0.34)
	**1000**	0.65 (0.012)	0.59 (0.008)	0.47 (0.007)	0.31 (0.27-0.32)
	**10,000**	0.57 (0.010)	0.58 (0.010)	0.47 (0.009)	0.32 (0.29-0.34)
	**IM**	0.55 (0.009)	0.56 (0.010)	0.46 (0.006)	0.29 (0.28-0.32)
**RM**	**100**	0.73 (0.021)	0.46 (0.024)	0.42 (0.015)	0.2 (0.15-0.37)
	**1000**	0.40 (0.050)	0.37(0.036)	0.36 (0.031)	0.12 (0.06-0.22)
**Polygenic**		0.39 (0.013)	0.40(0.012)	0.45 (0.012)	0.29 (0.27-0.32)

^1 ^Heritability was estimated using the REML method assuming the animal model.

The accuracy of the gBLUP method was less dependent on the various genetic models. gBLUP performed as well as Bayes B when variation was controlled by the infinitesimal model. It also performed competitively when variation was controlled by common variants under the QTL models, but the accuracy of breeding value prediction under the QTL models was lower than that achieved by Bayes B. Similarly under the RM model, gBLUP did not predict genetic values as accurately as Bayes B. However it was significantly better than traditional BLUP under the QM scenarios, the infinitesimal model and the RM with 100 rare variants and it also tended to be more accurate under the RM with 1,000 rare variants. When genetic variation was controlled by QTL with large, moderate or small effects, traditional BLUP was the least accurate method to predict breeding values. However, under the traditional polygenic model in reference population 1, BLUP was the most effective method to predict breeding values.

The accuracy of predicting breeding values significantly decreased for both genomic evaluation methods when animals became less related (using reference populations 2 and 3) (Tables 2 and 3). With large QTL effects, prediction accuracy persisted over many generations when using Bayes B to predict breeding values. Similarly gBLUP was also able to predict a small proportion of the variation in breeding values in unrelated individuals. Using reference populations 2 and 3, traditional BLUP was unable to accurately predict breeding values of animals in the test set when the reference population consisted of distantly related animals. However, when variation was modelled as the traditional polygenic model based on pedigree relationships, all of the methods were unable to estimate breeding values for the distantly related individuals.

**Table 2:** The average accuracy of breeding value estimates (±SE) in the test set obtained from three methods of analysis of reference population 2 with 60,000 SNPs and different genetic models

Model	No. QTL	Bayes B	gBLUP	BLUP
**QM**	**100**	0.77 (0.014)	0.37 (0.018)	0.01 (0.011)
	**1000**	0.49 (0.015)	0.38 (0.018)	0.08 (0.018)
	**10,000**	0.33 (0.013)	0.32 (0.010)	0.02 (0.007)
	**IM**	0.35 (0.012)	0.36 (0.015)	0.09 (0.009)
				
**RM**	**100**	0.67 (0.022)	0.26 (0.027)	0.01 (0.021)
	**1000**	0.31 (0.044)	0.25 (0.022)	0.04 (0.015)
				
**Polygenic**		-0.01 (0.017)	0.00 (0.010)	0.07 (0.009)

**Table 3:** The average accuracy of breeding value estimates (±SE) in the test set obtained from three methods of analysis of reference population 3 with 60,000 SNPs and different genetic models

Model	No. QTL	Bayes B	gBLUP	BLUP
**QM**	**100**	0.77 (0.021)	0.33 (0.011)	0.00 (0.000)
	**1000**	0.47 (0.014)	0.34 (0.017)	0.00 (0.000)
	**10,000**	0.32 (0.012)	0.31 (0.010)	0.00 (0.000)
	**IM**	0.32 (0.015)	0.3 (0.017)	0.00 (0.000)
				
**RM**	**100**	0.63 (0.033)	0.21 (0.021)	0.00 (0.000)
	**1000**	0.25 (0.049)	0.19 (0.023)	0.00 (0.000)
				
**Polygenic**		0.00 (0.012)	-0.01 (0.010)	0.00 (0.000)

**Figure 1:**
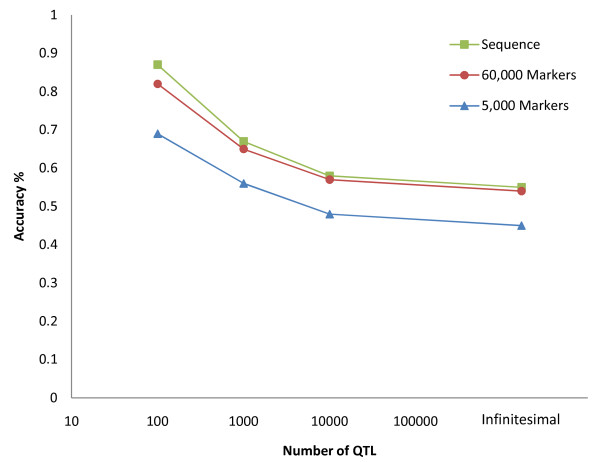
**The effect of the number of QTL and marker density on the accuracy of estimating breeding values in the test set using Bayes B (reference population 1)**.

The accuracy of estimating breeding values was higher when marker density was increased to whole-genome SNP sequence data (Table 4). When comparing Tables 1 and 2 with Table 4, the largest gains were observed when sequence information was used in both of the 100 QTL and 1,000 QTL models. Similarly, sequence data increased the ability of Bayes B to predict breeding values after many generations (reference population 2), increasing the accuracy by 5% for the 1,000 QTL model. Figure 1 illustrates that as the number of QTL increased, the accuracy advantage of using this sequence data decreased. Indeed when 10,000 QTL controlled genetic variation, the accuracy of prediction only increased by 1 percent from 0.57 using 60,000 markers to 0.58 using SNP sequence data and when the variation was controlled by the infinitesimal model there was no significant difference between 60,000 markers and sequence data. Similarly, the inclusion of sequence information had very little effect on the accuracy of prediction using gBLUP under all simulated models of variation.

**Table 4:** Accuracy of the estimated breeding values (±SE) using SNP sequence data using two different methods and two alternative reference populations

			Method	
	**No. QTL**	**Reference population**	**Bayes B**	**gBLUP**

**QTL**	**100**	1	0.87 (0.009)	0.58 (0.014)
	**1000**	1	0.67 (0.012)	0.60 (0.017)
	**10,000**	1	0.58 (0.013)	0.58 (0.015)
	**IM**	1	0.54 (0.015)	0.55 (0.012)
**QTL**	**100**	2	0.81 (0.021)	0.39 (0.020)
	**1000**	2	0.53 (0.017)	0.35 (0.013)
	**10,000**	2	0.38 (0.012)	0.34 (0.015)
	**IM**	2	0.34 (0.012)	0.35 (0.017)

## Discussion

We have found that the Bayes B method was the most accurate method to predict breeding values and was the most robust against changes to the model underlying genetic variation. Previously, Meuwissen et al. [1] and Habier et al. [9] have obtained similar results to those observed in this study, whereas Daetwyler et al. [17] reported that in some instances gBLUP predicted more accurate breeding values than Bayes B.

The current study has shown that even under infinitesimal assumptions when all SNP explain small amounts of variation, and even when there is an absence of detectable QTL effects, Bayes B will perform as well as gBLUP. A possible explanation is that under the IM and the traditional polygenic model, the Bayes B method will use information from a number of selected SNPs, and although the effects may be poorly estimated and a random set of markers is used, the resulting prediction is similar to gBLUP. Habier et al. [9] have shown that gBLUP is equivalent to a mixed model fitting all marker loci with equal variance (RR BLUP) and a genomic relationship matrix based on a subset of markers, as selected in the Bayes B method, may be a reasonable approximation of the genomic relationship matrix based on all markers [18]. In essence, the Bayes B method may estimate the relationships of animals based on a weighted subset of SNP, with weights derived from the variance explained at each locus.

In the analysis using Bayes B, π was set to 0.95 for all models and keeping this constant may have influenced the results for Bayes B. Given that many QTL had small effects in the 10,000 QTL model and in the infinitesimal model, it would have been very difficult to estimate the QTL that had non-zero effect sizes. There has been some recent work by Habier et al. [19] regarding the estimation of π using Bayesian methods (referred to as Bayes Cπ) where π is jointly estimated in the analysis. However, there is little empirical evidence about the estimation of π when using the Bayes B method. The Bayes B analysis used in this study also required the genetic variance for the trait to be provided and in this case, we used the true genetic variance. This may have biased the results to favour Bayes B; however, the estimated genetic variance obtained from REML was very similar to the true genetic variance and this estimated variance can be used in the Bayes B analysis when the true genetic variance is unknown.

The extent of the differences between gBLUP and Bayes B was largely dependent on the model of genetic variation used to simulate the underlying variation. Similarly to Meuwissen et al. [1], high accuracies were observed when genetic values were predicted under the QM with few QTL having large effects. This model favoured the Bayes B approach and both GS methods were able to predict genetic values accurately over the different reference population scenarios. However, the accuracies achieved for the 100 QTL model are rarely observed when GS is used to predict breeding values in 'real' populations of this size (reference populations of 2,000 animals) and accuracies are commonly closer to 0.5 [20]. Moreover, results from dairy cattle data analysis show that gBLUP and Bayes B achieve very similar accuracies for most traits [21,14], as seen when more than 1,000 QTL were simulated. This suggests that in many cases, the model of variation in real populations may be controlled by many genes and behave somewhat like the model with many small QTL effects controlling variation.

The size and distribution of the QTL effects controlled the effectiveness of both GS methods. Given that all QTL effects in the RM and QM were sampled from a gamma distribution, there were fewer QTL actually responsible for large proportions of the genetic variance. In the 100 QTL model, the top 10 QTL explained 80% of the genetic variance and the largest QTL explained 25% of the variation. For the 1,000 QTL model, the largest QTL explained 5% of the genetic variation and the top 20 QTL explained 50% of the genetic variation. In the 10,000 QTL model, the largest QTL explained 1% of the variation and the top 100 QTL explained 30% of the variation. For the traditional polygenic model, no QTL were simulated, therefore both methods relied on estimates of pedigree relationships to accurately estimate breeding values.

Results from the simulated traditional polygenic model were also somewhat unrealistic, as there was no link between genotypes and phenotypes other than pedigree information. This bias towards pedigree information allowed traditional BLUP to outperform the GS methods. However, this model was useful to show that Bayes B also uses pedigree information to explain a proportion of breeding value in absence of any detectable QTL.

The results of the RM appeared to be highly variable, and a low accuracy was found especially for the gBLUP and BLUP methods. The estimates of heritability (Table 1) were highly variable and generally lower than under the QM, IM and polygenic models, resulting in lower accuracy of prediction. As a consequence of all variants being rare and with relatively high allele substitution effects, changes in the frequency of these alleles had a large effect on the overall genetic variance in the population. These low allele frequencies of QTL in generation 1 made it easy to "lose" variation due to drift under the RM which, led to large fluctuations in the results. This suggests that this model is unlikely to explain additive genetic variation, especially with all genetic variation being additive, as simulated in this study. However, in spite of all of the QTL being rare in this model, and therefore difficult to detect, Bayes B could predict a substantial amount of genetic variation with genetic markers, similar to the QM.

The accuracy of across-line or across-breed prediction can depend on the similarity between different populations or the extent of the divergence between two populations [22,23]. When using Bayes B, the estimation of breeding values for individuals that were many generations apart or across different lines may be possible when variation is controlled by a small number of QTL with large effects. However, as the number of QTL increases this ability to predict breeding values decreases. Although gBLUP does not predict the breeding values for these unrelated individuals as accurately as Bayes B, it still relies on QTL information to better predict the relationships between animals, since it is able to predict a proportion of breeding value in both reference populations 2 and 3 under the IM, whereas under the polygenic model this accuracy was zero. A larger divergence between breeds and limited LD across the two populations is expected to lead to less accurate across-breed prediction of breeding values from genomic data [23].

The overall prediction of breeding values rely on the degree of relationship between the predicted individuals and those in the reference population because the less related the predicted individuals were to those in the reference population, the lower the accuracy of prediction. This has important implications for breeding programs. If there are QTL with large effects, then accurate predictions may persist over generations, but long term predictions may not be as accurate when variation is controlled by a larger number of genes. Therefore, the larger the number of small genes controlling variation the more important it is that animals included in the reference population are genetically more related to selection candidates. Additionally continuous updating of the reference population will be needed to maintain an accurate level of genomic prediction over generations.

Much debate has arisen around the effect of marker density on GS prediction accuracy. Low density marker panels may be cheaper and more cost effective for use in livestock prediction. Higher marker densities are expected to be more accurate, with sequence data expected to give the highest accuracy. For example, Yang et al. [6] have suggested that in human studies a low amount of LD may be a cause of inaccurate estimates of genetic values for lower density SNP panels. In our study, we used a population with a much lower effective size than in humans (therefore having a higher LD). A 5k SNP panel appeared to give significantly lower accuracy of breeding value, likely due to insufficient LD, with such large distances between SNPs. However, it appeared that with this simulated effective population size (Ne of 100), most of the LD is accounted for by 60,000 markers, and only a very small increase in accuracy was achieved when using sequence data. Results from reference population 2 showed that, when predicting many generations ahead, i.e. as LD decreases, the advantage of using sequence data increases.

The additional value of using sequence data over 60k markers in increasing accuracy of genomic breeding values was directly related to the size of simulated QTL effects. It was expected that sequence data would be very accurate as all QTL genotypes were included in the data and LD was no longer limiting the accuracy of the prediction. Meuwissen and Goddard [24] have found very high accuracies of up to 0.97 under a model similar to our 100 QTL model. Our study shows that a lower accuracy is likely when there are more QTL each with a smaller effect, as Bayes B is unable to estimate smaller QTL effects accurately, as shown when all SNP control variation (IM). This suggests that if a trait is highly polygenic, then the additional value of using sequence data will be smaller in terms of increased accuracy of estimated breeding values. When marker density is high enough to account for LD, the accuracy of genomic selection will be largely limited by the size of the reference population.

## Conclusions

Our results suggest that Bayes B is a superior method to gBLUP to estimate breeding values from genomic data. The method accurately estimates breeding values under a model with large QTL effects, but even if QTL with larger effects are not evident, it gives a similar accuracy of prediction to those obtained using gBLUP. The underlying model of genetic variation greatly affects the predictive ability of genomic selection methods, and their superiority over BLUP prediction depends on the presence of QTL effects. The use of sequence data will outperform the less dense marker panels as long as QTL effects can be estimated accurately. However the size and distribution of QTL effects will still greatly influence the effectiveness of using sequence data in genomic prediction. If a trait is more polygenic, then the inclusion of sequence information may not increase the accuracy of breeding values unless the reference population is very large.

## Competing interests

The authors declare that they have no competing interests.

## Authors' contributions

SAC performed the simulation, analyses and drafted the manuscript. JHJW, JMH, and SAC conceived and designed the experiment. All authors have read and approved the final manuscript.

## References

[B1] Meuwissen THE Hayes BJ Goddard ME Prediction of total genetic value using genome-wide dense marker maps Genetics 2001 157 1819 1829 11290733 10.1093/genetics/157.4.1819 PMC1461589

[B2] Habier D Tetens J Seefried FR Lichtner P Thaller G The impact of genetic relationship information on genomic breeding values in German Holstein cattle Genet Sel Evol 2010 42 5 10.1186/1297-9686-42-5 20170500 PMC2838754

[B3] Muir WM Comparison of genomic and traditional BLUP-estimated breeding value accuracy and selection response under alternative trait and genomic parameters J Anim Breed Genet 2007 124 342 355 10.1111/j.1439-0388.2007.00700.x 18076471

[B4] Goddard ME Hayes BJ McPartlan H Chamberlain AJ Can the same genetic markers be used in multiple breeds? Proceedings of the 8th World Congress on Genetics Applied to Livestock Production: August 13-18, 2006, Brazil. CD-ROM communication no. 22-16

[B5] Maher B Personal genomes: the case of the missing heritability Nature 2008 456 18 21 18987709 10.1038/456018a

[B6] Yang J Benyamin B McEvoy BP Gordon SD Henders AK Nyholt DR Madden PA Heath AC Martin NG Montgomery GW Goddard ME Visscher PM Common SNPs explain a large proportion of the heritability for human height Nature Genetics 2010 42 565 571 10.1038/ng.608 20562875 PMC3232052

[B7] Fearnhead NS Wilding JL Winney B Tonks S Bartlett S Bicknell DC Tomlinson IP Mortensen NJ Bodmer WF Multiple rare variants in different genes account for multifactorial inherited susceptibility to colorectal adenomas Proc Natl Acad Sci, USA 2004 101 15992 15997 10.1073/pnas.0407187101 15520370 PMC528777

[B8] Fisher RA The correlation between relatives on the supposition of mendelian inheritance Trans R Soc Edin 1918 52 399 433

[B9] Habier D Fernando RL Dekkers JCM The impact of genetic relationship information on genome-assisted breeding values Genetics 2007 177 2389 2397 18073436 10.1534/genetics.107.081190 PMC2219482

[B10] Maki-Tanila A Kennedy BW Mixed model methodology under genetic models with a small number of additive and non-additive loci Proceedings of the 3rd World Congress on Genetics Applied to Livestock Production: Lincoln 1986 443 448

[B11] Chen GK Marjoram P Wall JD Fast and flexible simulation of DNA sequence data Genome Res 2009 19 136 142 19029539 10.1101/gr.083634.108 PMC2612967

[B12] Villa-Angulo R Matukumalli LK Gill CA Choi J Van Tassell CP Grefenstette JJ High-resolution haplotype block structure in the cattle genome BMC Genetics 2009 10 19 19393054 10.1186/1471-2156-10-19 PMC2684545

[B13] Hickey JM Tier B AlphaBayes: user manual 2009 UNE, Australia

[B14] Hayes BJ Bowman PJ Chamberlain AC Goddard ME Invited review: Genomic selection in dairy cattle: Progress and challenges J Dairy Sci 2009 92 433 443 10.3168/jds.2008-1646 19164653

[B15] VanRaden PM Efficient methods to compute genomic predictions J Dairy Sci 2008 91 4414 4423 10.3168/jds.2007-0980 18946147

[B16] Gilmour AR Gogel BJ Cullis BR Thompson R ASReml User Guide Release 3.0 Hemel Hempstead: VSN International Ltd 2009

[B17] Daetwyler HD Pong-Wong R Villanueva B Woolliams JA The impact of genetic architecture on genome-wide evaluation methods Genetics 2010 185 1021 1031 10.1534/genetics.110.116855 20407128 PMC2907189

[B18] Rolf MM Taylor JF Schnabel RD McKay SD McClure MC Northcutt SL Kerley MS Weaber RL Impact of reduced marker set estimation of genomic relationship matrices on genomic selection for feed efficiency in Angus cattle BMC Genetics 2010 11 24 20403185 10.1186/1471-2156-11-24 PMC2868785

[B19] Habier D Fernando RL Kizilkaya K Garrick DJ Extension of the Bayesian Alphabet for Genomic Selection Proceedings of the 9th Congress on Genetics Applied to Livestock Production: 1-6 August 2010; Leipzig 2010 468

[B20] Moser G Tier B Crump RE Khatkar MS Raadsma HW A comparison of five methods to predict genomic breeding values of dairy bulls from genome-wide SNP markers Genet Sel Evol 2009 41 56 10.1186/1297-9686-41-56 20043835 PMC2814805

[B21] VanRaden PM Van Tassell CP Wiggans GR Sonstegard TS Schnabel RD Taylor JF Schenkel F Invited review: Reliability of genomic predictions for North American Holstein bulls J Dairy Sci 2009 92 16 24 10.3168/jds.2008-1514 19109259

[B22] Goddard ME Genomic selection: Prediction of accuracy and maximisation of long term response Genetica 2009 136 245 257 10.1007/s10709-008-9308-0 18704696

[B23] de Roos APW Hayes BJ Goddard ME Reliability of genomic breeding values across multiple populations Genetics 2009 183 1545 1553 10.1534/genetics.109.104935 19822733 PMC2787438

[B24] Meuwissen THE Goddard ME Accurate prediction of genetic values for complex traits by whole-genome resequencing Genetics 2010 185 623 31 10.1534/genetics.110.116590 20308278 PMC2881142

